# Profile of Geohelminth Eggs, Cysts, and Oocysts of Protozoans Contaminating the Soils of Ten Primary Schools in Dschang, West Cameroon

**DOI:** 10.1155/2017/1534675

**Published:** 2017-09-17

**Authors:** Vanessa Rosine Nkouayep, Blandine Ngatou Tchakounté, Josué Wabo Poné

**Affiliations:** Research Unit of Biology and Applied Ecology, Department of Animal Biology, Faculty of Science, University of Dschang, P.O. Box 067, Dschang, Cameroon

## Abstract

Helminthiasis and protozoans infections have been recognized as an important public health problem. The aim of the present study was to screen soil samples collected from 10 primary schools in the city of Dschang for the presence of soil-transmitted helminth eggs, cysts, and oocysts of protozoans. A total of 400 soil samples were collected around latrines, at playgrounds, and behind classrooms in each school. These samples were examined using the sucrose flotation method. From the result obtained, an overall contamination rate of 7.75% was observed. Five genera of nematodes (*Ascaris*,* Trichuris*,* Capillaria*,* Cooperia*, and hookworms) were identified, while neither cysts nor oocysts of protozoans were detected. The contamination rate and the number of species found were significantly different in wet season as compared to the dry season. During the rainy season, this rate was 12.5% with all the parasitic stages identified, while, in the dry season, the soil contamination rate was 3% with the presence of only two genera (*Ascaris* and* Trichuris*). This suggests that parasite infection may occur mainly in rainy season rather than in the dry season. The most common eggs were those of* Ascaris* with 2% and 5% contamination rates in the dry and rainy seasons, respectively. Also, the soils around latrines were more contaminated (11.9%) as compared to those collected behind classrooms (7.5%) and those at playground (2.5%). It was concluded that the pupils of these schools may have played a major role in the contamination of their environment. Thus, sanitary education, enforcement of basic rules of hygiene, and deworming remain a necessity in the entire population of the study area in general and in the schools in particular in order to prevent helminth infections and to ensure effective environmental health.

## 1. Introduction

Soil contamination by infective forms of intestinal parasites is the most important infection risk factor for both humans and animals. These parasites have been recognized as an important public health problem, particularly in developing countries [1], where adequate water and good sanitation are lacking. The commonest and well known of these parasites are hookworms (*Necator* and* Ancylostoma*), whipworm* (Trichuris)*, and the common roundworm* (Ascaris)* [2]. They are most prevalent in man and can also be found in animals. These parasites have similar epidemiological characteristics with a direct life cycle. Host contamination occurs via oral route through ingestion of infective embryonated eggs from contaminated soil, vegetables, and food products or via the percutaneous migration of infective L_3_ from the environment. Recent estimation suggests that they infect over 1 billion, 770 million, and 800 million people, respectively. These parasites have been shown to negatively impact the physical fitness and cognitive performance of the pupils [3]. Mature nematode eggs, cysts, and oocysts of protozoan parasites can remain viable in the soil for a long time depending on several factors such as climatic conditions, seasonal air temperatures, humidity or desiccation of soil, and exposure to sunlight [4]. Thus, contamination of soils with infective forms of parasites may be an important source of infection and constitutes a great risk factor for human infections, especially for small children aged below 12 years because of their vulnerability to nutritional deficiency and since they usually play within the grounds [5]. Studies conducted in various cities all over the world show variable prevalence of soil contamination with different parasite genera. But, in Cameroon, to the best of our knowledge, none or few epidemiological data are available about the rate of soil contamination. In the light of that, there is a need to survey the soils around latrines, at playgrounds, and behind classrooms of school children for the presence of parasites so as to take preventive measures to avoid the impact of the disease on children. The present study was therefore conducted to determine the profile of geohelminth eggs, cysts, and oocysts of protozoans in the soils of 10 primary schools in Dschang, Cameroon.

## 2. Materials and Methods

### 2.1. Study Area

**Figure 1:**
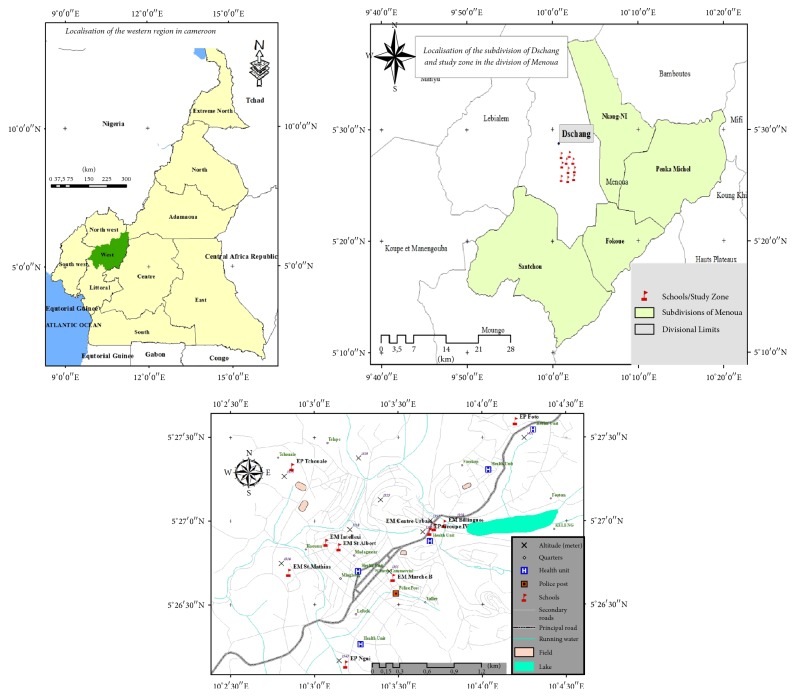
Study area and sampling points.* Source*: GPS Garmin; image satellite: QuickBird 2014, Dschang, Cameroon.

This study was carried out in Dschang (West Cameroon) (Figure 1). Dschang is located between latitude of 5°20′ north and longitude of 10°30′ west and at an altitude of about 1407 m. This city covers an area of 225 km^2^ and has a cool, mild climate (wet tropical) of the Equatorial Guinean type characterized by two seasons: a rainy season that runs from mid-March to mid-October and a dry season from November to February. Rainfall is unimodal with an annual height of 1809 mm. High precipitation is observed in August and September. Soils encountered in this city are hydromorphic and ferralitic soils.

### 2.2. Study Design

Ten schools of the city of Dschang were selected for this study based on their topographic position (plateaus, hills, and lowlands) (Figure 1). Thus, at plateaus' level, these schools were primary school of Foto (EP Foto), Tchouale primary school (EP Tchouale), and Saint-Mathias-Foréké kingdom nursery school (EM St Mathias). At the hills' level, schools were Center Urban nursery school (EM Centre Urbain), Group IV primary school (EP Groupe IV), and bilingual nursery school Dschang (EM Billingue); and at the lowlands' level, the schools were Ngui primary school (EP Ngui), Intellexi nursery school (EM Intellexi), Market B nursery school (EM Marché B), and Saint-Albert Anglo primary school (EP St Albert).

### 2.3. Sample Collection

400 soil samples were collected during two periods: August-September 2014 (rainy season) and January-February 2015 (dry season). During each season, soil samples were collected at three sites of each school: around latrines, at playgrounds, and behind classrooms. About 200 grams of soil dug at 3 cm from the ground was collected using a small hand shovel [6]. The soil samples were carried using a clean spoon and put in small plastic bags which were labeled with the date, the school name, and sample sites. The samples were kept in a bag and transported to the laboratory for parasitological analysis.

### 2.4. Survey Method

The samples were analyzed by the sucrose floatation technique as described by Uga et al. [7]. In the laboratory, each soil sample was sieved with a 150 *μ*m sieve to remove large particles. 2 grams of powder was weighed and put in a test tube and mixed with 8 ml of distilled water and then centrifuged at 1000 rpm for 5 min. After centrifugation, the supernatant was discarded and the pellet rediluted with 8 ml of sucrose solution (1.20). The mixture was vigorously shaken and centrifuged at 2000 rpm for 10 min. Finally, the interface and the upper layer obtained were collected and introduced into 3 test tubes until the formation of the upper meniscus. Three cover slips were carefully placed above the tubes. After 10 minutes, cover slips were removed carefully and placed on 3 slides and observed microscopically at 10x and 40x. Since cysts are denser, the pellet of each tube was shaken and one drop removed, placed on a slide using a pipette, and covered with a cover slip. Thereafter, a drop of 1% Lugol was subsequently placed on the edge of the cover slip and the preparation observed under a microscope at 40x magnification. The identification of eggs, cysts, and oocysts was done by differential diagnosis based on morphological criteria such as size, shape, nature of the shell, the number of cores, the number of blastomeres, and karyosome [8–10].

### 2.5. Statistical Analysis

Statistical analyses were performed using the Statistical Package for Social Sciences 20.0 (SPSS 20.0). The Chi-square test was used to compare soil contamination percentages. The significance level for all tests was *p* = 0.05.

## 3. Results

### 3.1. Parasites Identified

Out of the 400 soil samples examined, 31 were positive to nematodes eggs given overall prevalence of 7.75%. Neither cysts nor oocysts of protozoans were identified (Table 1). Five types of nematodes were identified in the soil samples. Among these samples, 14 (3.5%) were contaminated with* Ascaris* eggs, 8 (2%) with those of* Trichuris*, 6 (1.5%) with those of* Capillaria*, 2 (0.5%) with the eggs of* Cooperia*, and 1 (0.25%) with hookworm eggs.

### 3.2. Seasonal Variation of Soil Contamination and Sample Site-Related Prevalence

Table 2 shows the comparison of soil contamination between the dry season and rainy season. It appears that the soil samples collected in wet season were more contaminated, 25 (12.5%), as compared to those from the dry season, 6 (3%). Irrespective of the season, the highest prevalence was seen with* Ascaris*,* Trichuris*, and* Capillaria *eggs.

Table 3 shows the prevalence of nematode eggs in soil with respect to the sample sites. We observed that soil samples collected around latrines were more contaminated by nematode eggs (11.9%) followed by those behind classrooms (7.5%) and those from playgrounds (2.5%).* Ascaris* eggs were highly present (3.5%) as compared to those of* Trichuris* (2%) and* Capillaria* (1.5%).

### 3.3. Soil Contamination Rate-Related School

**Figure 2:**
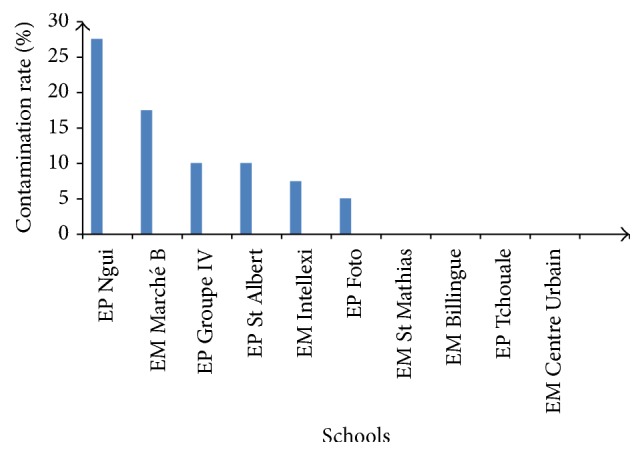
Percentages of soil contamination in different schools.

The percentage of soil contamination related to school is shown in Figure 2. The soil samples collected in Ngui site have the highest rate (27.5%) of contamination with parasitic stages followed by those from Market B nursery school (17.5%) and those of Intellexi nursery school (7.5%). The samples from Group IV and St Albert had the same rate (10%) of contamination and those of Foto were the least contaminated (5%). The soil samples for other schools were free from contamination.

## 4. Discussion

A total number of 400 samples of soils obtained from 10 different schools were examined for geohelminth eggs and protozoan cysts and oocysts. We found that the contamination of soil samples was due to 5 genera of nematodes eggs:* Ascaris, Trichuris, Capillaria, Cooperia, *and hookworms. This finding is similar to studies reported in Nigeria [11, 12] and in other countries of the world. For example, the study carried out by Shrestha et al. [13] on the soil-transmitted helminths in Kathmandu, Nepal, has demonstrated the presence of nematodes eggs, namely,* Ascaris*,* Trichuris*, and hookworms. Besides these, they also observed the presence of* Toxocara* eggs,* Vampirolepis nana*, and* Taenia* embryophores which were not found in this work. The absence of the embryophore of* Vampirolepis nana* in the present study could be justified by the fact that this parasite is most prevalent in hot and dry country [14], whereas Dschang has a cool climate. For the absence of* Taenia* eggs, it can be justified by the fact that, in Dschang, there is an improvement in farming conditions with livestock. Other nematodes such as* Strongyloides* and pinworms have not been identified in any of the works mentioned above. In fact, it is the larvae of* Strongyloides* that are found in nature and this work has not dwelt on the study of larvae. In general, the prevalence of nematode eggs in different works is due to their resistance to environmental conditions and poor sanitary and hygienic conditions [15]. Uga et al. [16, 17], Stojcevic et al. [18], and Tavalla et al. [19] observed in their studies in Nepal, Croatia, and Tehran, respectively, the presence of protozoan cysts (*Eimeria*,* Isospora*,* Cryptosporidium*, and* Giardia*) which were not found in this work. This could be due to (1) the different techniques of analysis used and (2) the soil texture of Dschang. It is known that the soil texture can also affect the survival of cysts in the soil as demonstrated by Davies et al. [20].

*Ascaris *eggs were more frequent in soil samples (3.5%) followed by those of* Trichuris *(2%),* Capillaria* (1.5%),* Cooperia* (0.5%), and hookworms (0.25%). The high prevalence of* Ascaris *eggs in soil samples of the present study is similar to other observations reported elsewhere [11–13]. This can be explained by the fact that eggs of* Ascaris* have an inner shell layer of lipoprotein nature which makes them more resistant to harsh environmental conditions and air [21] compared to the eggs of other nematodes. Another reason is that* Ascaris *eggs can survive in adverse environmental conditions. It might also be due to the overdispersion of* Ascaris *eggs in the environment as a single female* Ascaris *lays relatively large number of eggs (200.000 eggs/day) [9]. Lower rate of soil contamination observed with* Trichuris *eggs might be due to their minimal dispersion as a single female* Trichuris *liberates relatively less numbers of eggs and also due to easy destruction of embryonated eggs by desiccation. One interesting finding in this study was the detection of* Capillaria* and* Cooperia* eggs of other animal origins which were not detected in other studies elsewhere. It might be due to the animal husbandry in the city. The absence or scarcity of hookworm eggs could be justified by their life cycle. In fact, after their release into the environment in 24 h–36 h, embryonation of the eggs takes place. The eggs hatch in the ground and the first moulting larvae (L_1_) generation is then released to give the infective larvae.

Soil contamination rate found in this study (7.75%) was lower than that reported elsewhere. This rate is lower than those obtained (11.25%, 53.6%, and 32%) by Debalke et al. [22], Chukwuma et al. [11], and Odoba et al. [12], respectively, on soils in some schools in Ethiopia, in primary schools of Ebene (Nigeria), and in some primary schools in Zaria (Nigeria). The differences in these rates could be due to climatic factors, socioeconomic and topographical characteristics, and soil texture which vary within countries. Indeed, these factors have an impact on the distribution of helminths in the environment [23]. The low contamination rate observed in this work could be due to the multiple annual deworming campaigns organized since few years in schools in Cameroon. This may reflect also the improvement in the living standards, literacy rate, health awareness, use of toilet, and others. We observed that Ngui primary school had the highest contamination rate (27.5%), significantly different (*p* < 0.05) from those registered in other schools. According to Yerima and Van Ranst [24], topography influences the environmental conditions of a place. Ngui primary school is located in lowland characterized by relatively high humidity that favors the development of nematode eggs. By contrast, water flow is observed at the level of plateaus and hills that participate in soil leaching with the parasite eggs. This can partly justify the low level of contamination observed in schools located in high altitude (EM St Mathias, Bilingual EM, EM central, and EP Foto).

Around latrines, 11.9% of soil samples were more contaminated with the eggs of nematodes compared to the other two sampling points, that is, at playgrounds (2.5%) and behind classrooms (7.5%). This finding corroborates findings obtained by Chukwuma et al. [11] in Nigeria. Considering the fact that the eggs come from the school children, it clearly indicates that some school children usually defecate around/behind toilets and behind classrooms instead of using the toilet. These two points are privileged places for school children's contamination by soil-transmitted helminths.

Regarding the seasons, soil samples were relatively more contaminated during the wet season (12.5%) than during the dry season (3%). These results corroborate those obtained elsewhere [16, 18, 25, 26]. Actually, it is known that the development of the egg in the soil depends on several factors such as temperature (optimum: 20–30°C) and adequate soil moisture [27]. Thus, the observations can be explained by the fact that the rains create environmental and climatic conditions favorable for the survival of nematode eggs. However, these eggs are also resistant to extreme conditions. It is for this reason that few eggs of* Ascaris* and* Trichuris* were observed in the dry season.

## 5. Conclusion

The findings reported in the present study showed that the rate of soil contamination was low but it remains to be the most direct indicator of risk factor for children's health in schools of Dschang. In addition, daily awareness of the need to keep the environment healthy by adequate sanitation practices should be highlighted in order to avoid soil contamination by human intestinal parasites.

## Figures and Tables

**Table 1:** Contamination rate of soil samples with identified nematodes.

Nematodes	Number of contaminated samples	Contamination rate (%)
*Ascaris *	14	3.5
*Trichuris*	8	2
*Capillaria*	6	1.5
*Cooperia*	2	0.5
Hookworms	1	0.25
*Total*	*31*	*7.75*

**Table 2:** Type and frequency of nematode eggs recorded in rainy and dry seasons.

Nematodes	Seasons		Total *n* (%)
	Rainy season *n* (%)	Dry season *n* (%)	
*Ascaris*	10 (5)	4 (2)	14 (3.5)
*Trichuris*	6 (3)	2 (1)	8 (2)
*Capillaria*	6 (3)	0	6 (1.5)
*Cooperia *	2 (1)	0	2 (0.5)
Hookworms	1 (0.5)	0	1 (0.25)
*Total*	*25 (12.5)*	*6 (3)*	*31 (7.75)*

**Table 3:** Type and frequency of nematode eggs found in soil samples per sampling sites.

Nematodes	Sites			Total (*n* = 400) Frequency (%)
	Around latrines (*n* = 160) Frequency (%)	Playgrounds (*n* = 120) Frequency (%)	Behind classrooms (*n* = 120) Frequency (%)	
*Ascaris *	12 (7.5)^*∗*^	0	2 (1.7)	14 (3.5)
*Trichuris *	6 (4)	0	2 (1.7)	8 (2)
*Capillaria*	1 (0.7)	3 (2.5)	2 (1.7)	6 (1.5)
*Cooperia *	0	0	2 (1.7)^*∗*^	2 (0.5)
Hookworms	0	0	1 (0.9)^*∗*^	1 (0.25)
*Total*	*19 (11.9)*	*3 (2.5)*	*9 (7.5)*	*31 (7.75)*

^*∗*^Statistical significance (*p* < 0.05).
